# Bilateral Facial Paralysis and Deafness in a Child Treated for Acute Lymphoblastic Leukemia

**DOI:** 10.1155/2019/7126043

**Published:** 2019-11-06

**Authors:** Rafael V. Lucena, Yuri C. F. Fernandes, Débora B. Pazinatto, Rebecca C. K. Maunsell

**Affiliations:** Department of Otolaryngology Head and Neck, Faculty of Medical Sciences, State University of Campinas (UNICAMP), Campinas, SP, Brazil

## Abstract

Involvement of the ear and temporal bone in acute leukemias are uncommon. We report a case of atypical mastoiditis with bilateral facial paralysis in a child diagnosed with Acute Lymphoblastic Leukemia (ALL). A 20-month-old male child was diagnosed with ALL and developed otorrhea unresponsive to antimicrobial treatment during the first week of chemotherapy followed by hearing loss, loss of balance, and bilateral facial paralysis. A CT scan of the mastoids showed cortical erosion of the temporal bone and presence of soft tissue contents filling the mastoid cells and external auditory canal bilaterally. Mastoidectomy was performed to collect material for analysis. Histopathologic examination of the material revealed an active chronic inflammatory process, with a moderate amount of plasma cells. Chemotherapy was reintroduced 3 weeks after the surgical procedure, and progressive improvement of otorrhea and imbalance was noted. Grade III House–Brackmann peripheral facial paralysis persisted on 6-month follow-up, and the patient is in rehabilitation program.

## 1. Background

Acute lymphoblastic leukemia (ALL) accounts for 30% of all childhood malignancies. It involves malignant transformation and proliferation of immature lymphoid cells in the bone marrow, blood, or extramedullary sites [1]. Approximately 60% of cases occur in people under 20 years of age, with a peak between 2 and 5 years of age [2]. The WHO 2016 review establishes two major subtypes of the disease: B-cell acute lymphoblastic leukemia/lymphoma (most common) and T-cell acute lymphoblastic leukemia/lymphoma [3]. Clinical and laboratory presentation at diagnosis as well as genetic and biological aspects of leukemia cells are used as the main prognostic factors [4].

Involvement of the ear and temporal bone in acute leukemias is uncommon and may present as skin lesions, changes in the tympanic membrane, effusions in the middle ear, otitis media, hearing loss, or mastoiditis. Pathophysiology may be due to bleeding, leukemic infiltration, or opportunistic infection at these sites [5, 6]. Facial paralysis due to VII cranial pair involvement is very rare, with few cases described in the literature.

We report a case of atypical mastoiditis with bilateral peripheral facial paralysis in a child diagnosed with ALL.

## 2. Case Presentation

A 20-month-old male child was admitted to the emergency room with fever, associated with cutaneous-mucosal pallor, inappetence, irritability, and bruises in the lower limbs at the onset 11 days ago. He did not present any otological complaint, loss of balance, or signs of central nervous system involvement.

Laboratory analysis showed a white blood cell count of 79,300 × 10^9^/L with a differential of 8% lymphocytes and 92% blasts; hemoglobin was 7.9 g/dL, and platelet count was 79,000 × 10^9^/L. The patient referred to children's hospital for complementary exams. The myelogram revealed acute lymphoid leukemia (ALL), with 98% of lymphoblasts. Immunophenotyping was compatible with CD10 + cytoplasmic IgM + B-cell precursor acute lymphoblastic leukemia (pre-B ALL), karyotype 45, XY, -21. Cerebrospinal fluid (CSF) analysis did not show the presence of leukemic cells, and a computed tomography (CT) scan of the head at that time showed no cranial abnormality.

Chemotherapy treatment was initiated with the AIEOP-BFM 2009 protocol: prednisone, daunorrubicin, vincristin, peg-asparaginase, and intrathecal methotrexate [7]. On the 11th day of treatment, serosanguineous otorrhea was initiated first in the right ear and after two days in the left ear. On the 19th day of treatment, the patient presented fever. The hemoglobin was 6.2 mg/dL, platelets 4,000/dL, and white blood cell count was 750/dL. Blood and secretions from the middle ear were cultured, and both were positive for Pseudomonas sp. A new CSF analysis was performed and found a white blood cell count of 7/mm³ without leukemic cells, low glucose concentration, or increased protein. Meropenem and otological drops of ciprofloxacin were introduced.

Although fever resolved, the patient maintained serosanguineous otorrhea. On the 27th day of chemotherapy, the parents reported that the child was unable to smile or locate sounds, besides presenting difficulties walking. The patient was then referred to otorhinolaryngology. Otoscopy showed bilateral purulent otorrhea and an opacified, apparently bulging, tympanic membrane bilaterally. There was no spontaneous or semispontaneous nystagmus, but the child was unable to walk without support. Severe facial mimic dysfunction was noted bilaterally (House–Brackmann grade IV), with no other cranial nerve impairment. A complete blood count revealed a hemoglobin level of 6.9 mg/dL, platelet count of 286,000/dL, and white blood cell count of 15,890/dL with 56% of polymorphonuclear leukopcytes, 28% of lymphocytes, and 15% of monocytes.

**Figure 1:**
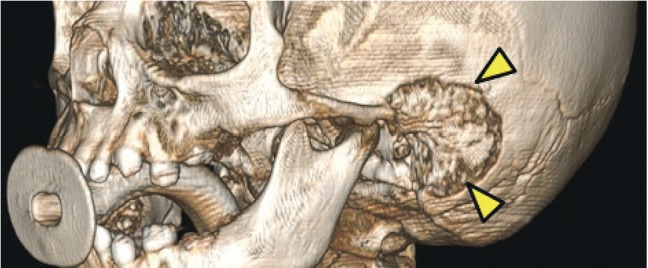
Cortical erosion of the temporal bone (yellow arrows) in a CT scan. 3D view.

**Figure 2:**
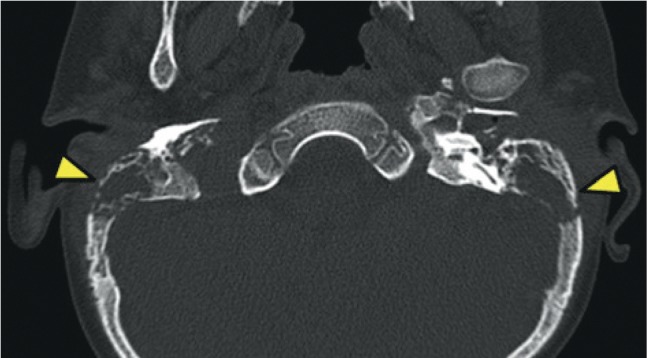
Cortical erosion of the temporal bone (yellow arrows) in a CT scan. Axial view.

A CT scan of the ear and mastoids showed bone erosion of the temporal bone and mastoid and presence of soft tissue contents filling the mastoid cells, involving the ossicular chain with extension to the external auditory canal in both ears (Figures 1 and 2).

**Figure 3:**
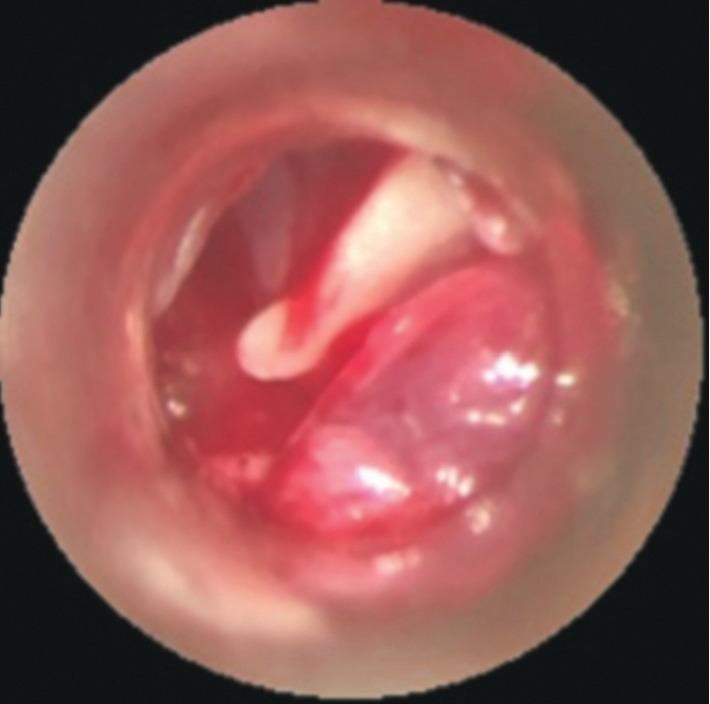
Micro-otoscopy showing fibroelastic tissue in the external auditory canal.

**Figure 4:**
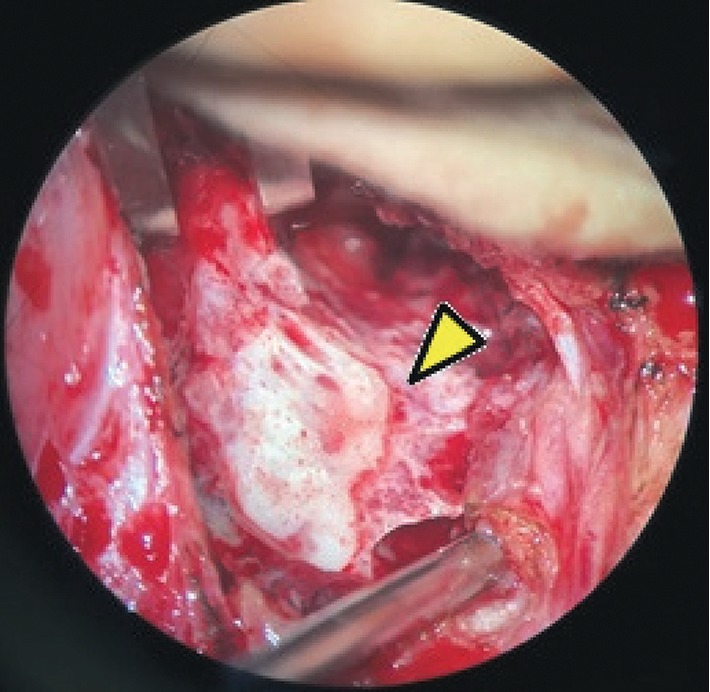
Mastoidectomy showing erosion of the cortical bone.

**Figure 5:**
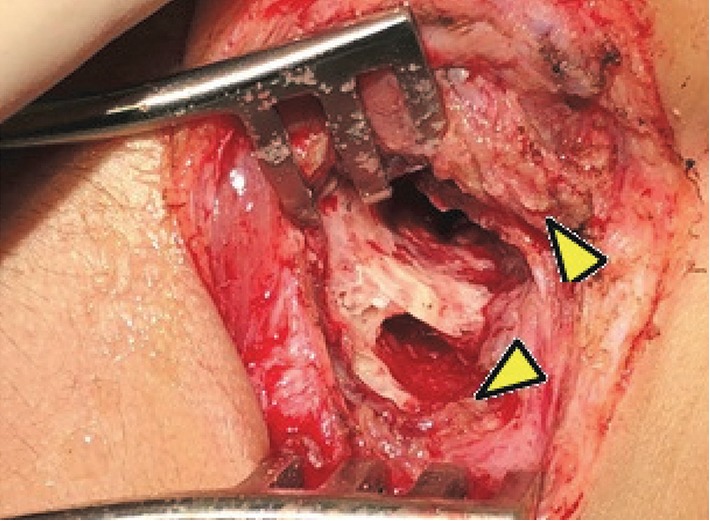
Tissue of infiltrative aspect in the temporal bone.

A right mastoidectomy and left tympanotomy were performed and a whitish fibroelastic friable lesion was found both in the mastoid extending to the tip of the mastoid and the middle ear with partial erosion of the posterior wall of the external ear canal. Large tympanic perforations were seen bilaterally with “hyperplasic” lesion filling the middle ear. The cortical part of the temporal bone and the underlying tissues were infiltrated in appearance (Figures 3–5). Histopathologic examination of the material revealed an active chronic inflammatory process, with a moderate amount of plasma cells and absence of blast cells.

The patient restarted chemotherapy after 3 weeks of the surgical procedure and progressively improved of balance complaints and otorrhea.

## 3. Outcome and Follow-Up

**Figure 6:**
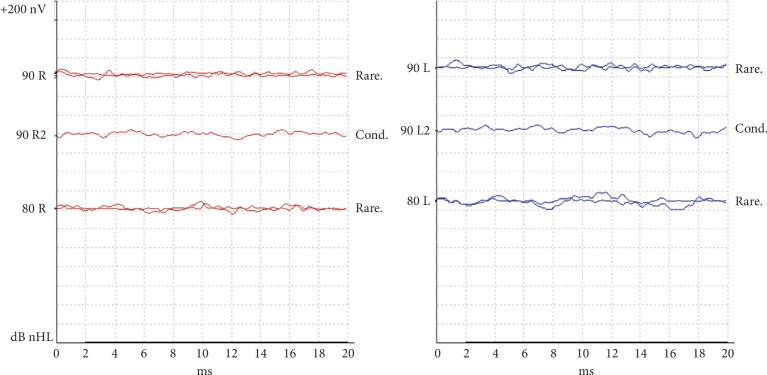
Absence of response on BERA at 90 dB.

At 2-month follow-up Brainstem Evoked Response Audiometry (BERA) with click and tone burst stimulation revealed severe bilateral sensorineural hearing loss with absence of response in 90 dB in both ears (Figure 6).

At a 6-month follow-up, the patient was in the maintenance phase of chemotherapy and presented mild improvement of the facial paralysis on the left side (grade III House–Brackmann). A new CT scan of the ears and mastoid showed no ossicular chain bilaterally, with apparent content of soft parts in the mastoid cells. He was referred to auditory rehabilitation program, but use of hearing aids has not shown recognizable improvement. No central nervous involvement of ALL has been identified.

## 4. Discussion

In the pioneer study by Druss in 1945, it was observed that patients with leukemia had a significant number of secondary otologic complications. He reviewed the medical records of 148 patients with different apresentations of leukemia and found that almost 17% experienced secondary complications such as hearing loss, nystagmus, vertigo, facial paralysis, and tympanomastoid infections [8].

In 1973, Paparella et al. conducted the first large study with temporal bones of leukemia patients. The authors analyzed 45 temporal bones of 25 patients with chronic and acute leukemia. They found that all clinical otologic problems occurred only in patients with acute form of leukemia, particularly in acute lymphocytic leukemia. The histopathological findings were leukemic infiltration, hemorrhage, and infection in middle ear more frequently than inner ear or external auditory canal [9].

Okura and Kaga in 1994 also described the histopathological findings of the middle ear in 19 patients with leukemia. He observed middle ear effusion in 8 of 35 ears analyzed, and in 4 of these ears effusion floating tumor cells were found. The study suggests that the major causes of middle ear effusion in patients with leukemia are infection related to immunological deficiency, obstruction of the Eustachian tube with infiltration of tumor cells, and a tendency to bleed due to hematological problems [10].

In our case, bilateral serosanguineous otorrhea associated with fever initially led to the suspicion of suppurative acute otitis media. Signs of an active bacterial infection with pus and local inflammatory reaction were not present at this time probably due to the absence of polymorphonuclear cells caused by leukemia and chemotherapy. The change in the aspect of otorrhea is associated with its maintenance even during treatment with antibiotic therapy and the appearance of other symptoms such as loss of balance and facial paralysis alerted to an atypical presentation of mastoiditis. Pediatric acute mastoiditis of noninfectious etiology is much rarer than true infectious mastoiditis. It should be suspected in every atypical, persistent, or recurrent case of acute mastoiditis [11]. According to Kontorinis, all cases of atypical mastoiditis must be conducted with routine laboratory investigation, imaging studies, and tissue biopsies.

The case presented by Todd and Bowman raises questions regarding the role, timing, and extent of surgery in atypical acute mastoiditis in leukemia patients. According to the author, surgery is appropriate when the patient's course indicates more bacteriologic and pathologic information is needed. When tumor is encountered in the ear and intraoperative histopathologic study is inconclusive or not available, the extent of surgery must be limited to obtaining sufficient tissue for histopathologic diagnosis, reducing tumor bulk (to facilitate the subsequent usefulness of radiation or chemotherapy) and preserving function of the ear [5]. In the present case, exploratory mastoidectomy was performed in the right ear to collect material and posterior decision-making considering the patient's underlying disease. Microscopy of the left ear revealed a lesion with similar and particularly atypical aspect for infectious otomastoiditis, and again the decision was to collect biopsy specimen only. As described in the literature, since diagnosis was not clear, the decision was not to decompress the facial nerve at the time [5].

Bilateral facial paralysis and profound sensorineural hearing loss presented by the patient may have occurred due to the facial and acoustic nerve involvement by leukemic infiltration or severe local inflammatory reaction triggered or not by infection. Bilateral facial paralysis is an unusual clinical entity that occurs in less than 1% of patients with facial paralysis. Bilateral facial paralysis is even more rare in children, and establishing its etiology can be challenging [12]. The case reports about acute lymphoblastic leukemia suggest a preponderance of T-cell leukemia for this complication [13]. Neurologic symptoms and meningeal signs should always be carefully investigated as 50% of leukemia children with central nervous system relapse can present with cranial nerve involvement, particularly of the facial nerve [12]. There are at least two possible explanations for the association of facial paralysis and leukemia: a direct infiltration of the nerve with leukemic cells and a common etiologic infectious factor such as Epstein–Barr virus or human T-cell lymphotropic virus. Neuropathy occurs due to compression and damage of the nerve and their vessels by infiltration of leukemic cells [14]. Although the histopathological analysis was not conclusive for leukemic infiltration of the mastoid, other multiple factors are probably associated in this case [15], such as local infection exacerbated by the depressed immune status, drug toxicity, hemorrhage, and local edema, making it very difficult to separate and define a single etiollogy. Partial improvement of facial paralysis in response to chemotherapy leads us to hypothesize that perineural infiltration may have occurred.

A database search of Medline, PubMed, BVS-BIREME, Web of Science, Embase, and SCOPUS, from 1984 to December 2018, was performed for studies that reported facial paralysis in child population with acute lymphoblastic or myeloid leukemia. The search terms used in database searches were “Facial Paralysis” AND “Precursor Cell Lymphoblastic Leukemia-Lymphoma” OR “Acute Myeloid Leukemia” AND “Infant” OR “Preschool” OR “Child” OR “Adolescent”. We found five case reports of children or adolescents with acute lymphoblastic leukemia presenting with bilateral facial paralysis. Two cases [14, 16] had bilateral facial paralysis as a first symptom of leukemic relapse in the central nervous system (CNS), and involvement of the middle ear with otological symptoms was present in only one. The chemotherapy treatment showed no improvement in both cases. Krishnamurthy et al. [17] reported a case of an infant with bilateral facial paralysis and ALL. He had performed bilateral tympanocentesis after a CT scan showing presumed bilateral mastoiditis unresponsive to antibiotics. Chemotherapy was initiated, and facial paralysis improved at a 6-month follow-up. Berger et al. [18] reported a case of an 8-year-old boy who presented otalgia and was unsuccessfully treated for otitis media. He presented a soft tissue edema with severe stenosis of bilateral external auditory canal and developed a bilateral facial paralysis House–Brackman grade VI. Similarly, to the present case, a CT scan of the temporal bone revealed opacification of the external ear canal and mastoid. His audiogram presented a mild conductive hearing loss. Further investigation led to B-cell ALL diagnosis. Different from ours, no biopsy was performed, and the patient's left side facial paralysis improved after 1 month of chemotherapy.

A concise review of the literature is presented in Table 1.

## 5. Learning Points

The patient discussed in this study presented unusual symptoms rarely and diversely reported in the literature. The involvement of the temporal bone in acute leukemias in children, although rare, should always be remembered either as initial symptomatology or during the course of chemotherapy when apparently major inflammatory response associated or not to infection may result in complications. Patients who present atypical mastoiditis, symptoms of imbalance or facial paralysis (uni- or bilateral) should be referred to the otorhinolaryngologist. A broad investigation and active discussion amongst the treating oncologist and otolaryngologist, with laboratory tests, imaging exams, and if necessary surgical intervention, are necessary in order to establish a diagnosis and provide the best treatment.

## Figures and Tables

**Table 1:** Literature review.

Study, year	No. of patients	Age of patients	Disease	Facial paralysis (FP) side	Otological surgery	Middle ear biopsy	Outcome of FP with chemotherapy
Todd and Bowman, 1984 [5]	1	13 yr	AML	Right	Yes	Granulocytic sarcoma	Complete resolution 6-month follow-up
Levy et al., 1986 [15]	1	5	AML	Right	Yes	Leukemic infiltration	Complete resolution
Ingram, 1990	9	ND	ALL	ND	No	—	ND
Zappia et al., 1990 [19]	1	6 yr	AML	Left	Yes	Granulocytic sarcoma	Complete resolution in 3-month follow-up
Juhn and Inoue, 1996 [20]	1	14 yr	T-cell ALL	Bilateral (alternately)	No	No	ND
Eser et al., 2001 [21]	1	14 yr	AML	AML	No	No	Complete resolution
Buyukavci et al., 2002 [13]	1	13 yr	T-cell ALL	Bilateral	No	No	Partial resolution
Krishnamurthy et al., 2002 [17]	1	11 mo	ALL	Bilateral	Yes	No	Improvement in 6-month follow-up
	1	11 mo	AML	Right	No	No	No improvement
	1	6 yr	ALL (Burkitt's)	Left	No	No	Improvement in 6-month follow-up
Antunes et al., 2004 [16]	1	18 yr	ALL	Bilateral	No	No	No improvement
Bilavsky, 2006	1	8 mo	AML	Right	No	No	ND
Lakhotia et al., 2015 [14]	1	15 yr	Pre-B-cell ALL	Bilateral	No	No	No improvement
Li et al., 2016 [22]	1	11 yr	T-cell LBL	Right	Yes	Lymphoma	Complete resolution
Berger et al., 2016 [18]	1	8 yr	B-cell ALL	Bilateral	Yes	No	Partial resolution
Young et al., 2016 [23]	1	2 yr	AML	Bilateral	No	No	Improvement in 5-month follow-up
Chiang et al., 2017 [24]	1	12 yr	T-cell ALL	Right	No	No	Complete resolution
Sagar et al., 2018 [25]	1	6 yr	AML	Right	Yes	Leukemic infiltration	Complete resolution

Abbreviations: ALL: acute lymphoblastic leukemia, AML: acute myeloid leukemia, LBL: lymphoblastic lymphoma, ND: no description.
